# Thermal Control Using Far-Infrared Irradiation for Producing Deglycosylated Bioactive Compounds from Korean Ginseng Leaves

**DOI:** 10.3390/molecules27154782

**Published:** 2022-07-26

**Authors:** Shucheng Duan, Jia Rui Liu, Xin Wang, Xue Mei Sun, Han Sheng Gong, Cheng Wu Jin, Seok Hyun Eom

**Affiliations:** 1 College of Food Engineering, Ludong University, Yantai 264025, China; dsc97@khu.ac.kr (S.D.); ljr19980227@163.com (J.R.L.); 17616196867@163.com (X.W.); xuemei0110@163.com (X.M.S.); hsgong_221@163.com (H.S.G.); 2 Department of Smart Farm Science, College of Life Sciences, Kyung Hee University, Yongin 17104, Korea

**Keywords:** ginseng leaf, far-infrared irradiation, polyphenols, ginsenosides, antioxidants, health benefits

## Abstract

Although ginseng leaf is a good source of health-beneficial phytochemicals, such as polyphenols and ginsenosides, few studies have focused on the variation in compounds and bioactivities during leaf thermal processing. The efficiency of far-infrared irradiation (FIR) between 160 °C and 200 °C on the deglycosylation of bioactive compounds in ginseng leaves was analyzed. FIR treatment significantly increased the total polyphenol content (TPC) and kaempferol production from panasenoside conversion. The highest content or conversion ratio was observed at 180 °C (FIR-180). Major ginsenoside contents gradually decreased as the FIR temperature increased, while minor ginsenoside contents significantly increased. FIR exhibited high efficiency to produce dehydrated minor ginsenosides, of which F4, Rg6, Rh4, Rk3, Rk1, and Rg5 increased to their highest levels at FIR-190, by 278-, 149-, 176-, 275-, 64-, and 81-fold, respectively. Moreover, significantly increased antioxidant activities were also observed in FIR-treated leaves, particularly FIR-180, mainly due to the breakage of phenolic polymers to release antioxidants. These results suggest that FIR treatment is a rapid and efficient processing method for producing various health-beneficial bioactive compounds from ginseng leaves. After 30 min of treatment without leaf burning, FIR-190 was the optimum temperature for producing minor ginsenosides, whereas FIR-180 was the optimum temperature for producing polyphenols and kaempferol. In addition, the results suggested that the antioxidant benefits of ginseng leaves are mainly due to polyphenols rather than ginsenosides.

## 1. Introduction

Ginseng (*Panax ginseng* Meyer) roots and their processed products are widely consumed because of their excellent health benefits, which are attributed to their bioactive ginsenosides [1]. Qualified ginseng roots require a relatively long cultivation period and are typically harvested between 4 and 6 years. Consequently, ginseng leaves are produced in large quantities annually and disposed of as waste at the end of the growing season. However, ginseng leaves possess similar pharmacological activities to the roots [2]. The distribution of ginsenosides in ginseng leaves has been investigated, which suggests that the leaves have similar ginsenoside compositions to the roots [3,4,5]. In addition, Chung et al. [6] reported an extremely high total phenolic content of ginseng leaves compared to the roots. Yin et al. [7] suggested that ginseng leaves contain higher total flavonoids compared to the roots, especially flavonol glycosides of panasenoside and kaempferol-3-O-glucoside. However, although ginseng leaves are potentially good sources for producing phytochemicals, especially polyphenols and ginsenosides, and have advantages in annual yield production, ginseng leaves have not been fully studied and used.

Thermal processing methods play a vital role in the global ginseng market. Numerous studies have shown significant processing results related to the deglycosylation pattern of ginsenosides and various health-beneficial biological activities, exhibiting bioconversion from major ginsenosides to minor ginsenosides, as well as antioxidant, antitumor, and anti-inflammatory effects [8,9,10,11]. Nevertheless, considerable efforts are required to reasonably use ginseng leaves, because few studies have focused on phytochemical variations, especially polyphenols, during dry-heat thermal processing.

Far-infrared irradiation (FIR) treatment is widely used in the food-processing industry because it is easily applicable in terms of fast and efficient heating of plant materials for inducing the decomposition of multi-chain molecular clusters [12]. Our previous studies have suggested that FIR is an efficient method for accelerating ginsenoside conversions in ginseng roots and leaves [10,13], which is significantly different from traditional steaming treatment at the same temperature [13]. An increase in the content of bioactive compounds, such as phenolic acids and flavonoids, by FIR has been reported in rice, angelica, gamguk flowers, grapes, and buckwheat sprouts [14,15,16,17,18]. However, there is still no detailed study related to defining an adequate FIR temperature for producing the maximum range of bioactive compounds from ginseng leaves.

When considering cost, source availability, and sustainability, ginseng leaves are a more appropriate choice for obtaining phytochemicals, especially polyphenols and ginsenosides, compared to their roots. The aim of this study was to investigate the effects of FIR treatment on the bioactive compounds (total phenolics, flavonoids, and ginsenosides) and human health benefits (antioxidant activities) of ginseng leaves. Our results will provide useful information for the further use and application of ginseng leaves in the health product industry and food science.

## 2. Results and Discussion

### 2.1. Effects of FIR Treatment on TPC in Ginseng Leaves

**Figure 1:**
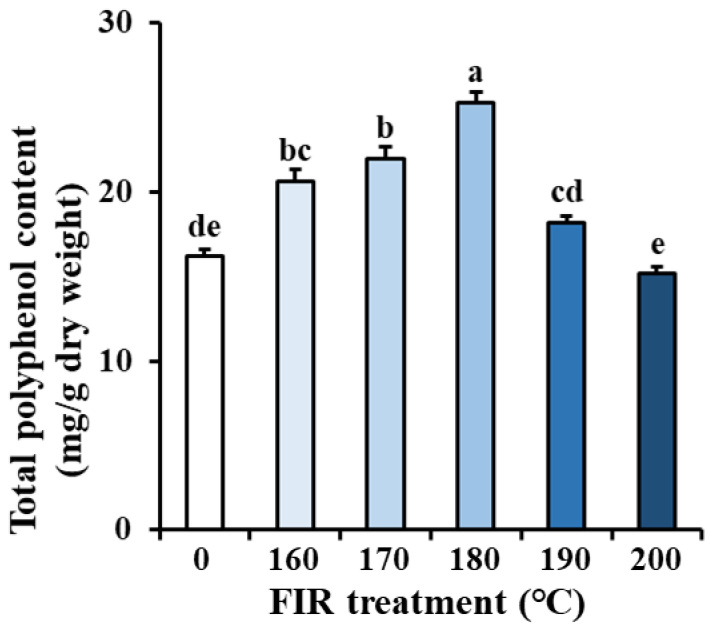
Changes in the total polyphenol content of ginseng leaves treated by FIR at different temperatures. The values are expressed as the mean with standard error (*n* = 3). Different letters (a–e) above the bar graphs indicate significant differences at *p* < 0.05 on Tukey’s HSD test.

The effects of FIR treatment on TPC in ginseng leaves are shown in Figure 1. As the FIR temperature increased, the TPC gradually increased up to 180 °C and then decreased. The highest TPC in ginseng leaves was 25.27 mg/g d.w. at FIR-180, which is about 1.56 times that of the untreated control. The lowest TPC (15.16 mg/g d.w.) was observed at FIR-200, with no significant difference being observed between FIR-200 and the untreated control. It can be concluded that suitable FIR treatment conditions can increase the TPC in ginseng leaves. The effect of FIR treatment on TPC improvement has also been reported in *Angelica gigas* Nakai, *Arachis hypogaea* L., *Camellia sinensis* var. sinensis, and *Hibiscus cannabinus* L. [15,19,20,21]. The increase in the TPC can be explained by the release of small polyphenols due to the breaking of molecular bonds in large polyphenols at high FIR temperatures [12,22]. However, relatively higher temperatures (FIR-190 and FIR-200) caused a decrease in the TPC in ginseng leaves compared to low temperatures (FIR-160, FIR-170, and FIR-180), which might be explained by the destruction of polyphenol structures by high FIR energy [18]. Based on our results, it can be concluded that 180 °C is a suitable temperature for FIR treatment for the processing of ginseng leaves to obtain more polyphenols. Polyphenols are an important phytochemical group in edible plants due to their various biological activities and health benefits, gaining increasingly more attention of researchers to investigate their content variation in plant tissues during different processing methods [23,24,25]. For ginseng, previous studies have mainly focused on their root polyphenol content changes during processing [26,27]. To the best of our knowledge, this is the first study to show the excellent effect of suitable FIR treatment on improving the TPC in ginseng leaves.

### 2.2. Effects of FIR Treatment on Panasenoside and Kaempferol Contents of Ginseng Leaves

**Figure 2:**
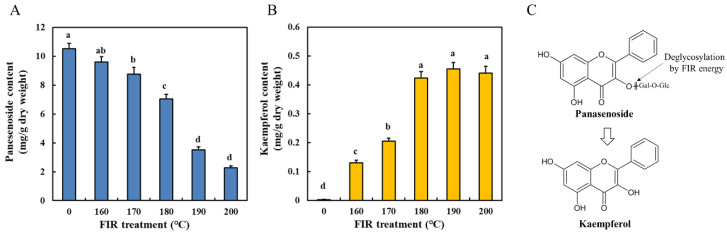
Changes in panasenoside (**A**) and kaempferol (**B**) contents and structural conversion pattern (**C**) from panasenoside to kaempferol in ginseng leaves treated by FIR at different temperatures. The values are expressed as the mean with standard error (*n* = 3). Different letters (a–d) above the bar graphs indicate significant differences at *p* < 0.05 on Tukey’s HSD test.

Panasenoside (kaempferol 3-*O*-glucosyl-(1-2)-galactoside) is a kaempferol glycoside and has been found to be the main flavonoid in ginseng leaves [7]. Figure 2A,B shows the variation in the contents of panasenoside and its aglycone (kaempferol) before and after FIR treatment. Before FIR treatment (0), ginseng leaves had 10.53 mg/g d.w. of panasenoside and only 0.03 mg/g d.w. of kaempferol. Similarly, Yin et al. [7] reported contents of 15.84 mg/g d.w. and 0.43 mg/g d.w of panasenoside and kaempferol, respectively, in 4-year-old ginseng leaves. After FIR treatment, a significant decrease in the panasenoside content of ginseng leaves was observed with increasing FIR temperature, with only 2.29 mg/g d.w. remaining in the leaves after FIR-200 treatment. In contrast, our results showed a significant increase in kaempferol. Its content increased sharply to 0.42 mg/g d.w. until FIR-180, which was 14.3 times that of the untreated control (0). The kaempferol content did not significantly increase at temperatures greater than 180 °C, such as FIR-190 and FIR-200.

According to the structures of panasenoside and kaempferol (Figure 2C), it is assumed that FIR energy causes a significant increase in the kaempferol content due to the deglycosylation of panasenoside. It has been suggested that FIR has the capacity to transfer heat energy, cleave covalent bonds, and liberate low-molecular-weight compounds [12,22]. However, the content of kaempferol did not significantly vary when the temperature exceeded 180 °C, despite the continuous decrease in the panasenoside content with increasing FIR temperature (Figure 2A,B). Furthermore, the increased aglycone content was not as high as that of panasenoside. The effects of FIR heat energy on ginseng leaf flavonoids were assumed in two situations according to temperature variation: (1) The relatively fast deglycosylation of panasenoside accompanied by the slow degradation of kaempferol occurred when FIR temperatures were below 180 °C, which explains the relatively slow decrease in the panasenoside content and the sharp increase in kaempferol, and (2) the rapid deglycosylation of panasenoside accompanied with the faster degradation of kaempferol when the FIR temperature exceeded 180 °C, which explains the rapid decrease in panasenoside and the lack of significant change in the kaempferol content. These assumptions were supported by Oliveira et al. [28]. They reported that the kaempferol content of *Poincianella pyramidalis* decreased significantly when the air inlet temperature was increased from 160 to 180 °C during spray-drying. Deng et al. [29] suggested that flavonol glycosides (kaempferol and quercetin derivatives) are degraded during the roasting process to correspond to an increase in aglycones in noni leaves (roasted at 175 °C from 10 to 60 min or roasted for 20 min from 100 to 250 °C, respectively). Moreover, FIR treatment usually provides higher energy during the material dry process compared to common dry processes under the same temperature control. In addition, it is expected that FIR treatment may lead to an intermediate compound by cleaving one glucoside of panasenoside rather than directly deglycosylating two glucosides to kaempferol. However, this intermediate compound was not detected in this study (data not shown). This result also supports our assumption. Previous studies on flavonoids in ginseng leaves have mainly focused on the types and contents of flavonoids in raw materials [7]. To the best of our knowledge, this is the first study to investigate flavonoid variation patterns in ginseng leaves during processing. According to our results, FIR treatment significantly increases the content of kaempferol via the deglycosylation of panasenoside (Figure 2), which has been found to possess higher bioactivities, such as antitumor, antioxidant, and anti-inflammatory activities, compared to its glycosides [30]. Thus, it can be concluded that FIR treatment (FIR-180) is a promising method for processing ginseng leaves to obtain more kaempferol. Here, it is also important to note that leaf burning was observed when the FIR temperature exceeded 200 °C during the 30 min treatment, suggesting that higher FIR temperatures are risky for ginseng leaf processing.

### 2.3. Effects of FIR Treatment on Ginsenoside Contents of Ginseng Leaves

**Figure 3:**
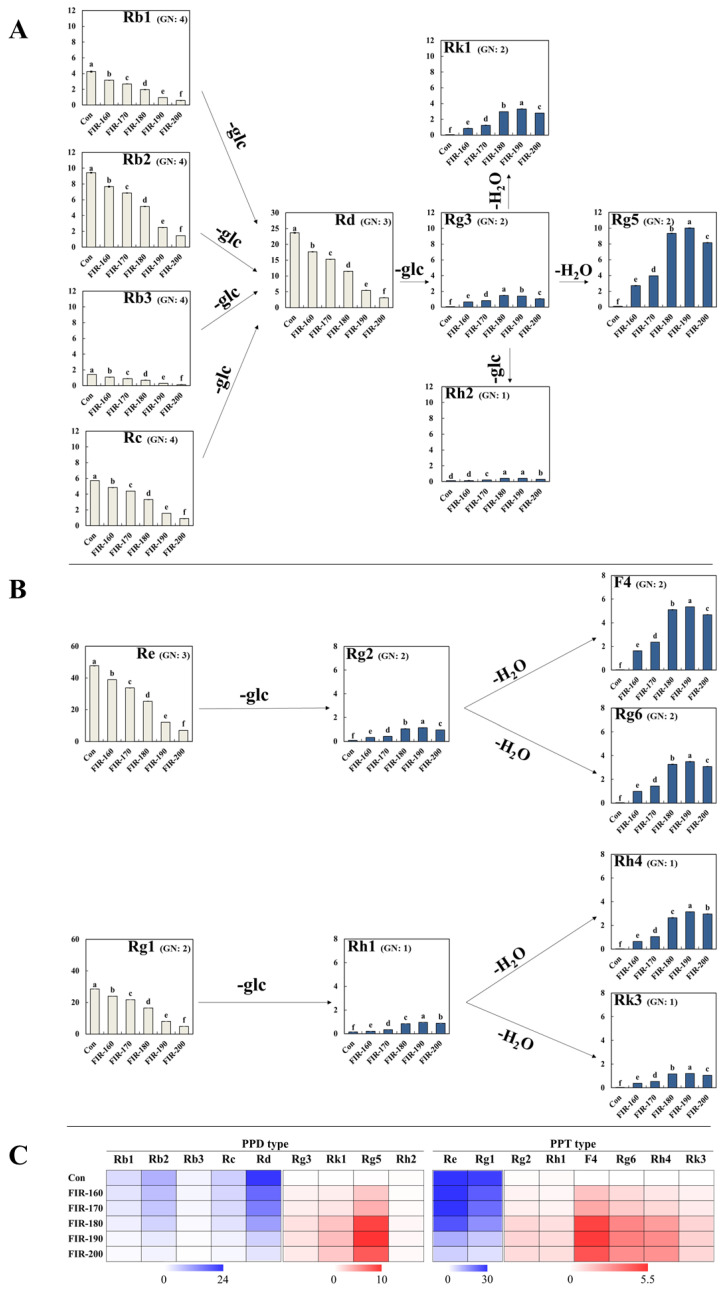
Flow schemes of the conversion patterns of PPD type (**A**) and PPT type (**B**) of ginsenosides in ginseng leaves treated by FIR at different temperatures. A heat map (**C**) of 17 kinds of ginsenoside changes in ginseng leaves treated by FIR at different temperatures. The unit of ginsenoside content is mg/g dry weight. GN indicates the glycosyl number attached on ginsenoisde. The values in bar graphs are expressed as the mean with standard error (*n* = 3). Different letters (a–f) above the bar graphs indicate significant differences at *p* < 0.05 on Tukey’s HSD test.

Ginsenosides can be roughly divided into two types according to the position and amount of glycol groups in the glycosides: (1) protopanaxadiol (PPD) type and (2) protopanaxatriol (PPT) type [8]. Here, the variations in nine PPD-type and eight PPT-type ginsenosides in ginseng leaves during FIR treatment were qualified and quantified using HPLC, respectively (Figure 3 and Figure S1, and Table S1).

#### 2.3.1. PPD-Type Ginsenosides

The effects of FIR treatment on the transformation of PPD-type ginsenosides in ginseng leaves are shown in Figure 3A,C, and Table S1. Overall, FIR treatment reduced the major ginsenoside content and increased the minor ginsenoside content. In detail, the ginsenosides Rb1 (4.25 mg/g d.w.), Rb2 (9.41 mg/g d.w.), Rb3 (1.42 mg/g d.w.), Rc (5.72 mg/g d.w.), and Rd (23.65 mg/g d.w.) were the predominant compounds in untreated ginseng leaves, whereas their contents gradually decreased after FIR treatment (Figure 3, light yellow). Each of the aforementioned ginsenosides showed a relatively low amount at FIR-200, with 0.58, 1.45, 0.13, 0.90, and 3.11 mg/g d.w. of Rb1, Rb2, Rb3, Rc, and Rd being observed, respectively. In contrast, non-FIR-treated ginseng leaves only had small amounts of minor ginsenosides (Figure 3, blue column), such as Rg3 (0.07 mg/g d.w.), Rk1 (0.04 mg/g d.w.), Rg5 (0.10 mg/g d.w.), and Rh2 (0.12 mg/g d.w.), whereas they significantly increased after FIR treatment. The ginsenoside Rg3 increased to its highest amount at FIR-180, presenting a 22-fold (1.49 mg/g d.w.) higher content than the untreated control. The highest amounts of Rk1, Rg5, and Rh2 were observed at FIR-190, which increased 77-fold (3.30 mg/g d.w. Rk1), 100-fold (10.01 mg/g d.w. Rg5), and 3-fold (0.41 mg/g d.w. Rh2), respectively, compared to the non-FIR-treated control.

FIR treatment significantly increased the content of minor PPD-type ginsenosides (Rg3, Rk1, Rg5, and Rh2) in ginseng leaves by achieving the deglycosylation and dehydration of the major ginsenosides. The major ginsenosides Rb1, Rb2, Rb3, Rc, and Rd had their glycosyl residue(s) at C-20 removed, causing a significant increase in the content of the minor ginsenoside Rg3. Furthermore, Rg3 was then converted to Rk1 and Rg5 via dehydration at C-20 and C-21/C-22 or to Rh2 by deglycosylation at C-3. Similar results have been reported for ginseng roots and observed in the ginsenoside variation patterns after different thermal processing methods, such as microwave heating, puffing, and steaming [9,31,32]. However, in our results, it is important to point out that most of the Rg3 was further converted to the dehydrated type (Rk1 and Rg5) instead of the deglycosylated type (Rh2). According to the carbenium ion mechanism [33], the deglycosylation of ginsenosides at C-20 is easy than at other positions because it can generate tertiary carbenium ions with more stability. Then, the active tertiary alcohol is further eliminated during thermal processing. In addition, Zaitsev’s rule can potentially give the reason why more Rg5 is enhanced than Rk1 during FIR treatment [34,35]. Our previous study suggested that 10 min of FIR treatment (between 60 and 120 °C) significantly increases the major ginsenoside (Rb1, Rb2, Rc, Rd) content of ginseng leaves, whereas it has no effect on minor ginsenosides (Rg3, Rh2) [13]. These results indicate that both the temperature and time of FIR treatment are important factors affecting ginsenoside variation in ginseng leaves. In addition, Chen et al. [36] reported that polar PPD-type ginsenosides (Rb1, Rb2, Rb3, Rc, and Rd) can be converted to Rg3, F2, Rh2, C-K, PPD, and Rk2 during the creation of black ginseng leaves. Thus, it is important to select suitable processing methods in consideration of the final products before processing ginseng leaves. Compared to FIR-190, a decreased pattern of each PPD-type minor ginsenoside was observed in FIR-200-treated ginseng leaves, which can be explained by the destruction of ginsenoside structures by high FIR energy.

#### 2.3.2. PPT-Type Ginsenosides

Re (47.71 mg/g d.w.) and Rg1 (28.47 mg/g d.w.) were the major PPT-type ginsenosides (Figure 3B,C, and Table S1) found in non-FIR-treated ginseng leaves. During FIR treatment, the Re and Rg1 contents gradually decreased as the FIR temperature increased, exhibiting 85% and 83% reductions at FIR-200, respectively. However, the content of minor ginsenosides, Rg2, Rh1, F4, Rg6, Rh4, and Rk3, significantly increased as the FIR temperature increased, presenting their highest amounts at FIR-190. Hundred-fold increases in most minor ginsenosides described above were found at FIR-190, namely 319-fold (5.34 mg/g d.w.) in F4, 169-fold (3.48 mg/g d.w.) in Rg6, 187-fold (3.14 mg/g d.w.) in Rh4, and 314-fold (1.21 mg/g d.w.) in Rk3 compared to the untreated control (less than 0.03 mg/g d.w. of each ginsenoside). The increases in Rg2 and Rh1 at FIR-190 were relatively small, exhibiting 8.07- and 3.11-fold increases compared to the untreated control, respectively.

The conversion patterns of the PPT-type ginsenosides are shown in Figure 3B. The increase in the minor ginsenoside Rg2 was as a result of the glycosyl residue at C-20 of the major ginsenoside Re being detached due to high FIR energy [8,22]. As we mentioned in Section 2.3.1, the fact that the glycosyl residue at C-20 was easily deglycosylated than at C-6 can be explained by the carbenium ion mechanism [33]. Rg2 was then converted to F4 via dehydration between C-20 and C-22 positions or to Rg6 via dehydration between C-20 and C-21 positions. Similar results were reported by Kim et al. [11] using standard ginsenosides. The standard ginsenoside Re was deglycosylated and transformed to the less polar ginsenosides Rg2, F4, and Rg6 after heat processing at 120 °C. Likewise, the deglycosylation of Rg1 at C-20 caused an increase in the Rh1 content, followed by an increase in Rk3 and Rh4, resulting from the dehydration of Rh1 between C-20 and C-21/C-22. A similar result was reported by Hwang et al. [31]. They showed the variation in Rg1, Rh1, Rh4, and Rk3 in white ginseng heated at different temperatures at 20 MPa for 2 h. In our results, relatively higher amounts of F4 and Rg6 were transformed compared to Rh4 and Rk3. Differently, Chen et al. [36] investigated ginsenoside changes in black ginseng leaf products and suggested that the degradation of Re and Rg1 to minor ginsenosides, such as Rk3, Rh4, and PPT, is the main transforming PPT-type pattern. This can be explained by the different progressions of thermal processing (methods, time, temperature, etc.) affecting different substances [13,37,38,39,40,41], as Rk3 and Rh4 are the deglycosylated products of Rg6 and F4 at C-6, respectively. Ginsenoside mutations in ginseng leaves during heat treatment have not been widely studied in the literature, especially in terms of dry-heat treatment. In our results, it is important to point out that FIR treatment (especially FIR-190) significantly increased the content of each dehydrated minor ginsenoside compared to unprocessed ginseng leaves (Figure 3C). The moderate FIR energy that showed high dehydration efficiency at C-20 instead of deglycosylation at C-6 was considered a response to the significant increased minor ginsenosides F4, Rg6, Rh4, and Rk3. A decreased pattern of each minor ginsenoside at FIR-200 compared to those at FIR-190 was also observed in our results, which may be due to ginsenoside degradation caused by excessive FIR energy.

These results suggest that a higher FIR temperature is more advantageous for ginseng leaf processing and indicate that 190 °C is a suitable temperature. It is also recommended for obtaining more dehydrated minor ginsenosides based on the processing conditions. Compared to major ginsenosides, minor ginsenosides show stronger or even new biological activities [42], so numerous studies have been devoted to promote the transformation of major ginsenosides into profitable ginsenosides through thermal processing [31,43]. Although our previous results have shown that FIR treatment can promote the conversion of major ginsenosides to minor ginsenosides in ginseng roots and leaves [10,17], this study optimized the processing technology and significantly improved the conversion efficiency in ginseng leaves.

### 2.4. Effects of FIR Treatment on Antioxidant Activities of Ginseng Leaves

The effects of FIR treatment on the antioxidant activities of ginseng leaves are shown in Table 1. Non-FIR-processed ginseng leaves (0; IC50 = 2.33) showed relative lower DPPH free-radical-scavenging ability compared to FIR-treated leaves, especially FIR-180 (IC50 = 1.52; Table 1.) Similar to the variations in DPPH radical-scavenging ability, ABTS radical-scavenging ability initially increased from FIR-160 to FIR-180 and then decreased (Table 1). These patterns show a distinct indication that appropriate FIR treatment can significantly improve antioxidant activities of ginseng leaves. Similar results were also observed in *Oriza sativa* L. [14], *Camellia sinensis* var. sinensis [20], and *Hibiscus cannabinus* L. [21], where FIR treatment increased antioxidant activities compared to non-treated controls.

In our results, extremely consistent variation patterns were exhibited between antioxidant activities and the TPC (Figure 1 and Table 1), which suggested that the antioxidant activities of ginseng leaves are most likely contributed by polyphenols rather than other compounds. The reason why stronger antioxidant activities in ginseng leaves were exhibited at FIR-160 to FIR-180 may be because FIR accelerates the release of antioxidants or leads to the transformation of certain substances to stronger-antioxidant-activity compounds, such as breakage of phenolic polymers [12,17,21]. The broken polyphenols exhibit better antioxidant activities. However, the reason why patterns of antioxidant activities decreased at higher FIR temperatures, FIR-190 to FIR-200, may be explained by the destruction of phenolic polymers and the degradation of simple formed phenolics. The degradation of phenolics may have been increasingly accelerated at temperatures higher than 180 °C in this study. It has been reported that ginseng leaves show stronger antioxidant activity compared to the roots [6,40]. In addition, several studies have evaluated the effects of different types of thermal processing on ginseng leaf antioxidant activities [43,44]. However, to the best of our knowledge, this is the first study to investigate the effect of FIR treatment on the antioxidant activity of ginseng leaves. Proper application of FIR can be considered a good method of improving the antioxidant activities and health benefits of herbal plants by increasing active polyphenols.

## 3. Materials and Methods

### 3.1. Chemicals

Organic solvents (HPLC grade) were purchased from Merck KGaA (Darmstadt, Germany). Folin–Ciocalteu reagent was purchased from Wako Pure Chemicals (Osaka, Japan). The standard compounds of panasenoside and kaempferol were purchased from Sigma Chemical Co. (St. Louis, MO, USA). Pure ginsenoside standards (98%) were purchased from ChromaDex (Santa Ana, CA, USA) and Ambo Institute (Daejeon, Korea).

### 3.2. Sample Collection and FIR Treatment

Six-year-old Korean ginseng leaves were used, which were collected from a ginseng farm in Wonju, Korea. Intact and undamaged leaves were washed with distilled water and then wiped with gauze. Fresh ginseng leaves were fully dried in an oven at 50 °C for 24 h and then ground using a grinder. The powder was meshed using a 200 μm sieve to obtain a uniform particle size of the powder. The powder was divided into two portions: (1) non-FIR-treated control (Con; 0) and (2) FIR treatments in an FIR dryer (HKD-10; Korea Energy Technology, Seoul, Korea) for 30 min at 160 °C (FIR-160), 170 °C (FIR-170), 180 °C (FIR-180), 190 °C (FIR-190), and 200 °C (FIR-200).

### 3.3. Bioactive Compound Analysis

#### 3.3.1. Sample Extraction

First, 2 g of each sample was weighed and added to 100 mL of 80% methanol solution (*v/v*). Sample extraction was performed in a shaking incubator for 24 h at 30 °C. After extraction, the solution was centrifuged (05PR-22 centrifuge, Hitachi, Tokyo, Japan) at 500× *g* for 10 min at room temperature. The supernatants were gathered after centrifugation and filtered through Whatman No. 42 filter paper (Whatman Inc., Clifton, NJ, USA). The filtrate was concentrated using a vacuum rotary evaporator (Eyela Co., Tokyo, Japan) at 40 °C. The samples were freeze-dried in a vacuum freeze-dryer (Christ Alpaha 1–4, Germany). Finally, the samples were stored in a refrigerator at −20 °C for subsequent experiments.

#### 3.3.2. Determination of TPC

The TPC was determined by the Folin–Ciocalteu method as follows: (1) Mix 1.9 mL of distilled water and 1.0 mL of Folin–Ciocalteu reagent in a tube, (2) prepare 0.1 mL of the sample solution (2 mg/mL, dissolved by 80% methanol) and add it to the tube, and (3) add 1.0 mL of 20% Na_2_CO_3_. The reaction mixture was incubated for 2 h at 25 °C. After incubation, the absorbance of the sample was recorded at 765 nm. The results were expressed as milligrams of gallic acid equivalents (GAE) per gram of dry weight (d.w.).

#### 3.3.3. High-Performance Liquid Chromatography (HPLC) Analysis of Panasenoside and Kaempferol

Panasenoside and kaempferol contents of ginseng leaves were determined using HPLC. The equipment was an HPLC system (CBM-20A; Shimadzu Co, Ltd., Kyoto, Japan) with 2 gradient pump systems (LC-20AT; Shimadzu, Japan), an auto sample injector (SIL-20A; Shimadzu), a UV detector (SPD-20A; Shimadzu), and a column oven (CTO-20A; Shimadzu). Each sample extract (0.1 mg) was dissolved in 1 mL of 80% methanol (*v*/*v*) and filtered through a 0.22 μm membrane filter before sample injection into the HPLC system. HPLC separation was performed on an Inertsil ODS-SP C18 column (250 mm × 4.6 mm, 5 µm; GL Sciences, Tokyo, Japan). The injection volume of samples was 10 μL. The gradient running phase was programmed with the combination of solvent A (water with 0.1% trifluoroacetic acid) and solvent B (acetonitrile), in which solvent B was sequentially increased from 14% to 18% (0 to 10 min), 18% to 30% (10 to 20 min), 30% to 60% (20 to 30 min), 60% to 65% (30 to 33 min), 65% to 100% (33 to 40 min), and 100% to 100% (40 to 50 min) and then finally adjusted from 100% to 14% (50 to 65 min). The operating temperature was set at 35 °C. The flow rate of the mobile phase was kept at 1.0 mL per min. The detector was set at 355 nm for monitoring panasenoside and kaempferol.

#### 3.3.4. HPLC analysis of Ginsenosides

The ginsenoside contents were determined by HPLC, the same equipment as mentioned before in Section 3.3.3. The prepared sample solution was filtered through a 0.22 μm membrane filter. The injection volume was 10 μL. A Kinetex C18 column (100 mm × 4.6 mm, 2.6 µm; Torrance, CA, USA) was used. The gradient running phase was programmed with the combination of solvent A (water) and solvent B (acetonitrile), in which solvent B was sequentially increased from 17% to 23% (0 to 30 min), 23% to 24% (30 to 35 min), 24% to 32% (35 to 45 min), 32% to 44% (45 to 48 min), 44% to 44% (48 to 52 min), 44% to 55% (52 to 65 min), 55% to 100% (65 to 85 min), and 100% to 100% (85 to 95 min) and finally adjusted from 100% to 17% (95 to 105 min). The operating temperature and flow rate were the same as those in the procedure mentioned in Section 3.3.3. The detector was set at 203 nm for monitoring ginsenosides.

### 3.4. Determination of Antioxidant Activities

The ginseng leaf powder (90 mg) was weighed, added to 30 mL of 80% methanol solution in a 50 mL tube, and extracted at 30 °C for 30 min with sonication. Then, the sample was centrifuged at 3500 r/min for 15 min. The supernatant was collected and filtered through a 0.22 μm membrane filter. The DPPH radical-scavenging activity was measured, as described by Eom et al. [17] with some modifications. Roughly, 1 mL of the sample solutions at different concentrations were mixed with 3 mL of DPPH solution. After the reaction for 30 min in the dark, the absorbance was measured at 517 nm. Next, the ABTS radical-scavenging ability was measured, as described by Lim et al. [45] with some modifications. Briefly, 0.5 mL of sample solutions at different concentrations were taken in a test tube and 5 mL of the prepared ABTS solution was added. After the reaction at room temperature for 10 min in the dark, the absorbance was measured at 734 nm. IC50 values denote the concentration of the sample, which is required to scavenge 50% of DPPH and ABTS free radicals.

### 3.5. Statistical Analysis

The data were analyzed statistically using SAS software (Enterprise Guide version 7.1; SAS Institute Inc., Cary, NC, USA). The data between non-FIR-treated control and FIR treatment groups were analyzed by one-way analysis of variance. The significance between experimental groups was evaluated using Tukey’s honestly significant difference (HSD) test at a *p* < 0.05 significance level.

## 4. Conclusions

This study studied in detail the effect of FIR on the representative health-beneficial compounds and antioxidant activities of ginseng leaves. Our results demonstrate that FIR treatment is a rapid and efficient method for producing deglycosylated bioactive compounds from ginseng leaves. FIR treatment at 180 °C is recommended to obtain more polyphoneols and kaempferol. However, the ideal FIR treatment temperature for producing deglycosylated minor ginsenosides, such as F4, Rg6, Rh4, Rk3, Rk1, and Rg5, is 190 °C. Both polyphenols and ginsenosides are compounds that are beneficial to human health, with different bioactivities that vary between individual compounds. However, with regard to the antioxidant activity of ginseng leaves, it seems to be mainly contributed by the highly accumulated polyphenols rather than ginsenosides. These findings will help the further use of ginseng leaves in health care products.

## Figures and Tables

**Table 1:** Changes in antioxidant activities (DPPH/ABTS radical-scavenging ability) in ginseng leaves treated by FIR at different temperatures.

FIR Treatment (°C)	IC50 (mg/mL)	
	DPPH Radical-Scavenging Ability	ABTS Radical-Scavenging Ability
0	2.33 ± 0.03 e	2.61 ± 0.04 e
160	1.72 ± 0.02 c	1.88 ± 0.04 c
170	1.61 ± 0.02 d	1.75 ± 0.02 b
180	1.52 ± 0.02 a	1.66 ± 0.02 a
190	1.98 ± 0.02 b	2.15 ± 0.03 d
200	2.47 ± 0.03 f	2.47 ± 0.03 e

The values are expressed as the mean ± SE (*n* = 3). Different letters within the same column indicate significant differences at *p* < 0.05 on Tukey’s HSD test.

## Data Availability

The data presented in this study are available in this article.

## Supplementary Materials

The following are available online at https://www.mdpi.com/article/10.3390/molecules27154782/s1, Figure S1. The chemical structures of analyzed ginsensides in this study. PPD, protopanaxadiol; PPT, protopanaxadiol; glc, β-D-glucose; arap, α-L-arabinopyranosyl; xyl, β-D-xylose; araf, α-L-arabinofuranosyl; Table S1. Changes of PPT and PPD ginsenoside contents (mg/g dry weight) in ginseng leaves treated to different FIR temperatures.
