# Supplementary material for: Thermal Control Using Far-Infrared Irradiation for Producing Deglycosylated Bioactive Compounds from Korean Ginseng Leaves
Source: Molecules. 2022 Jul 26;27(15):4782. doi: 10.3390/molecules27154782 (PMC9331281; doi:10.3390/molecules27154782)
Supplement: Supplementary file 1 [file molecules-27-04782-s001.zip › molecules-1814215-supplementary.pdf]

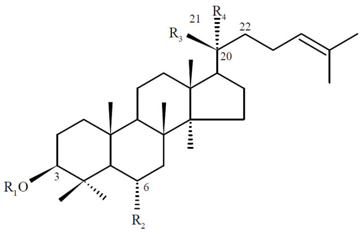

A

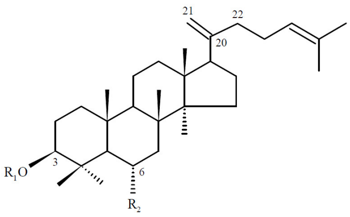

B

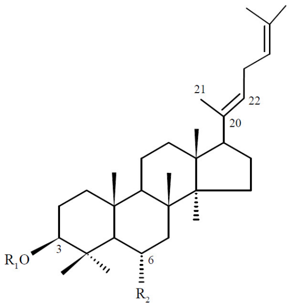

C

Structure	Type	Content	Ginsenoside	R_1	R_2	R_3	R_4
A	PPD	Major	Rb1	glc(2-1)glc	H	O-glc(6-1)glc	CH_3
			Rb2	glc(2-1)glc	H	O-glc(6-1)arap	CH_3
			Rb3	glc(2-1)glc	H	O-glc(6-1)xyl	CH_3
			Rc	glc(2-1)glc	H	O-glc(6-1)araf	CH_3
			Rd	glc(2-1)glc	H	O-glc	CH_3
		Minor	Rg3 (20S/R)	glc(2-1)glc	H	OH(CH_3)	CH_3(OH)
			Rh2	glc	H	OH	CH_3
	PPT	Major	Re	H	O-glc(2-1)rha	O-glc	CH_3
			Rg1	H	O-glc	O-glc	CH_3
		Minor	Rg2 (20S/R)	H	O-glc(2-1)rha	OH(CH_3)	CH_3(OH)
			Rh1	H	O-glc	OH	CH_3
B	PPD	Minor	Rk1	glc(2-1)glc	H		
	PPT	Minor	Rk3	H	O-glc		
			Rg6	H	O-glc(2-1)rha		
C	PPD	Minor	Rg5	glc(2-1)glc	H		
	PPT	Minor	Rh4	H	O-glc		
			F4	H	O-glc(2-1)rha		

Figure. S1. The chemical structures of analyzed ginsensides in this study. PPD, protopanaxadiol; PPT, protopanaxadiol; glc, β-D-glucose; arap, α-L-arabinopyranosyl; xyl, β-D-xylose; araf, α-L-arabinofuranosyl.

Table S1. Changes of PPT and PPD ginsenoside contents (mg/g dry weight) in ginseng leaves treated to different FIR temperatures.

**Ginsenoside**		**Treatment**					
		**Con**	**FIR-160**	**FIR-170**	**FIR-180**	**FIR-190**	**FIR-200**
**PPT**	Re	47.71± 0.13 a	38.94±0.02 b	33.72±0.05 c	25.27±0.05d	12.08±0.01 e	7.07±0.01 f
	Rg1	28.47±0.05 a	23.93±0.03 b	21.71±0.04 c	16.51±0.04 d	7.97±0.01 e	4.73±0.00 f
	Rg2	0.08±0.00 f	0.33±0.00 e	0.43±0.00 d	1.07±0.00 b	1.15±0.01 a	0.97±0.01 c
	Rh1	0.14±0.00 f	0.21±0.00 e	0.34±0.00 d	0.85±0.00 c	0.96±0.01 a	0.89±0.00 b
	F4	0.02±0.00 f	1.62±0.00 e	2.36±0.01 d	5.10±0.02 b	5.34±0.01 a	4.68±0.01 c
	Rg6	0.02±0.00 f	0.97±0.01 e	1.43±0.01 d	3.27±0.03 b	3.48±0.03 a	3.09±0.02 c
	Rh4	0.02±0.00 f	0.63±0.01 e	1.05±0.01 d	2.64±0.02 c	3.14±0.02 a	2.96±0.02 b
	Rk3	0.00±0.00 f	0.37±0.00 e	0.54±0.00 d	1.16±0.01 b	1.21±0.00 a	1.06±0.00 c
**PPD**	Rb1	4.25±0.06 a	3.15±0.01 b	2.68±0.01 c	1.97±0.02 d	0.95±0.01 e	0.58±0.02 f
	Rb2	9.41±0.06 a	7.66±0.06 b	6.85±0.02 c	5.16±0.02 d	2.49±0.02 e	1.45±0.01 f
	Rb3	1.42±0.01 a	1.08±0.01 b	0.90±0.00 c	0.69±0.01 d	0.30±0.00 e	0.13±0.01 f
	Rc	5.72±0.04 a	4.83±0.06 b	4.38±0.03 c	3.31±0.03 d	1.57±0.02 e	0.90±0.00 f
	Rd	23.65±0.14 a	17.67±0.06 b	15.30±0.04 c	11.47±0.03 d	5.44±0.01 e	3.11±0.01 f
	Rg3	0.07±0.00 f	0.65±0.01 e	0.83±0.00 d	1.49±0.00 a	1.40±0.01 b	1.06±0.01 c
	Rk1	0.04±0.00 f	0.85±0.00 e	1.24±0.01 d	2.96±0.01 b	3.30±0.01 a	2.78±0.00 c
	Rg5	0.10±0.01 f	2.72±0.03 e	3.96±0.01 d	9.32±0.03 b	10.01±0.03 a	8.15±0.02 c
	Rh2	0.12±0.00 d	0.14±0.00 d	0.20±0.01 c	0.41±0.00 a	0.41±0.00 a	0.30±0.00 b

Values are averages with standard errors from triplicate experiments. Different letters within the same line indicate significant differences at $p < 0.05$ on Tukey's HSD test.
